# Juglans Nigra, Black Walnut in Diphtheria

**Published:** 1881-04

**Authors:** 


Juglans Nigra, Black Walnut in Diphtheria.—Dr. C. R. S. Curtis, of Quincy, Ills., has an interesting article in the Boston

*Medical and Surgical Journal* on the use of a decoction of the leaves of the black walnut, to which, some times, in the worst cases, the green hulls were added to increase the strength of the decoction; as a wash or gargle to the throat in diphtheria. Occasionally some of the decoction was swallowed, also, with apparently good results

In about *thirty cases* treated with this decoction in conjunction with other remedies, all recovered. He thinks these results warrant a thorough trial by the profession. In which opinion we also agree.

